# Kerogen-rich rocks influence growth and composition of an anaerobic microbial community

**DOI:** 10.1038/s41598-026-42062-5

**Published:** 2026-03-08

**Authors:** Annemiek C. Waajen, Wessel de Wit, Mónica Sánchez-Román, John O. Edgar, Jon Telling, Charles S. Cockell

**Affiliations:** 1 UK Centre for Astrobiology, School of Physics and Astronomy, University of Edinburgh, Edinburgh, EH9 3FD UK; 2 Department of Earth Sciences, Vrije Universiteit Amsterdam, 1081 HZ Amsterdam, The Netherlands; 3 Faculty of Science, University of Amsterdam, 1098 XH Amsterdam, The Netherlands; 4 Mineralogy and Petrology Department, Sciences Faculty, University of Granada, Avenida de Fuentenueva S/N, 18071 Granada, Spain; 5 School of Natural and Environmental Sciences, Newcastle University, Newcastle, NE1 7RU UK

**Keywords:** Environmental microbiology, Biogeochemistry, Solid Earth sciences, Environmental sciences, Astrobiology

## Abstract

Kerogen, insoluble macromolecular organic matter in sedimentary rocks, is the most abundant form of organic carbon on Earth and plays a role in deep biosphere processes. It is classified into four types (I–IV) based on origin and chemical composition, yet its influence on microbial communities and carbon cycling remains poorly understood. In this study, we examined how kerogen-rich shales and coals, each containing a distinct kerogen type, shape anaerobic microbial community development and activity. CFU counts showed that kerogen types I and II did not significantly alter overall microbial abundance, while type III-rich rocks inhibited growth, and type IV-rich rocks enhanced it. 16S rRNA gene sequencing revealed that type II-rich rocks selectively enriched for Burkholderiaceae, whereas type IV-rich rocks promoted the proliferation of Cellulomonadaceae and Pleomorphomonadaceae. Gas chromatography showed that CO_2_ production occurred only in the presence of type II-rich rocks, likely driven by Burkholderiaceae activity. These findings suggest that kerogen structure and geochemical properties drive microbial community assembly and organic matter mobilization in the deep subsurface. Beyond Earth, kerogen type IV-like material is widespread in extraterrestrial environments. Our results indicate the enhancement of the habitability of these environments, offering new insights into the potential for life.

**Supplementary Information:**

The online version contains supplementary material available at 10.1038/s41598-026-42062-5.

## Introduction

The deep subsurface represents a vast reservoir of recalcitrant organic matter, with kerogen as its most abundant form. Kerogen is the insoluble macromolecular fraction of organic matter dispersed in sedimentary rocks, formed through the deposition and lithification of organic materials in aqueous environments^1, 2^. Accounting for an estimated 10^16^ tons of carbon, approximately twice the amount found in living biomass, kerogen plays a fundamental role in global carbon cycling, particularly in the deep biosphere^1, 3^. While traditionally regarded as geologically stable, recent studies suggest that kerogen-rich rocks may interact with microbial communities, potentially mobilizing organic matter and influencing deep subsurface ecosystem dynamics. The deep-subsurface is full of microbial life. It is estimated that half of all bacteria in the ocean live in the deep seabed^4^. Microbial life has been detected down to 2.5 km below the seafloor^5^ and in coal-beds of 20 million years old^6^. Coal-bearing sediment layers contain significantly higher cell concentrations than non-coal-bearing layers^5^, which implies that coal-bearing environments contain benefits for microbial life. This aligns with previous research showing that deeply buried black shales still act as active bioreactors 100 million years after deposition^7^.

**Fig. 1:**
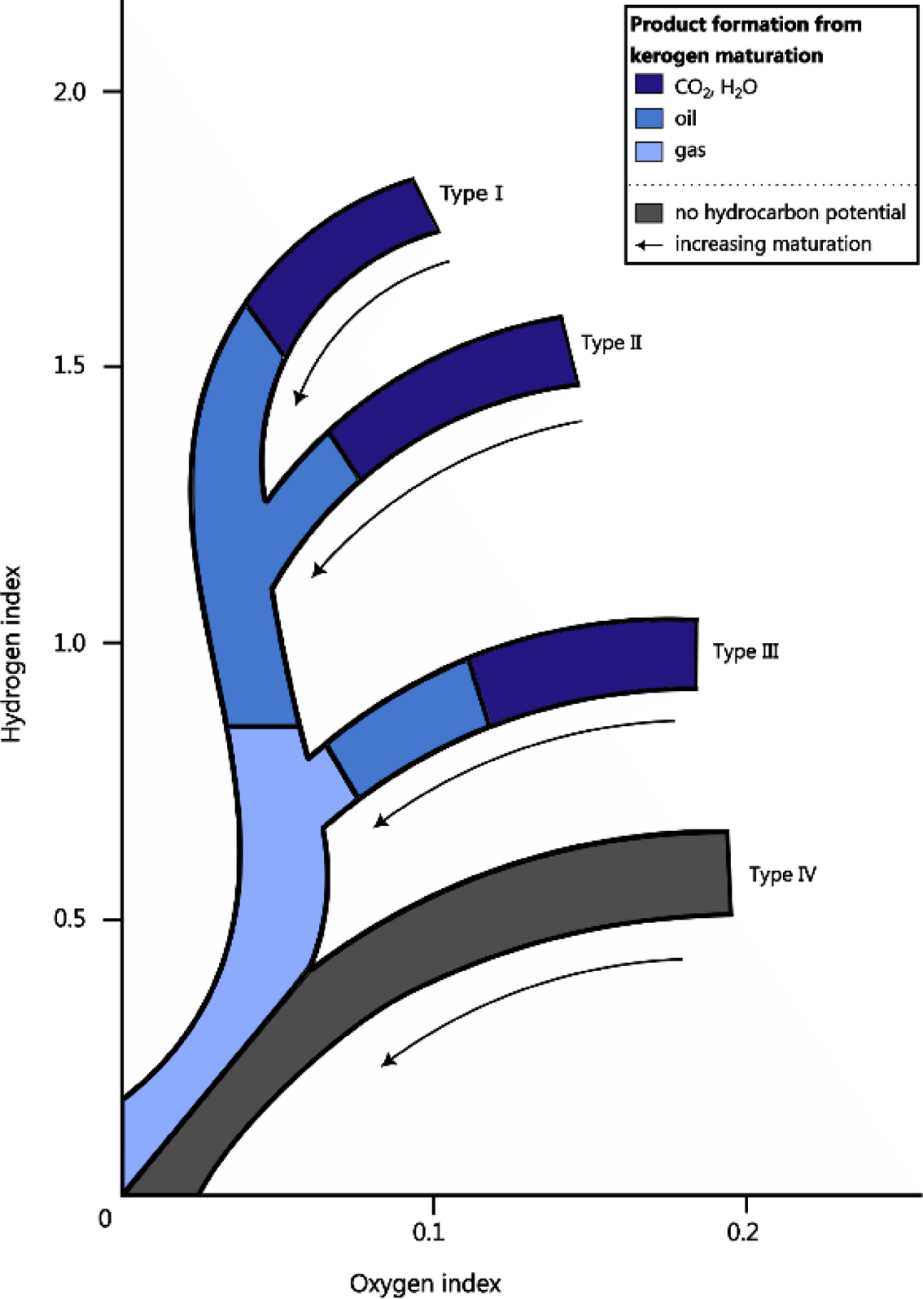
Van Krevelen diagram. The bands show the indication of the chemical differences between kerogen types and maturation based on the oxygen index (oxygen/carbon ratio) and hydrogen index (hydrogen/carbon ratio). Broader variances within the kerogen types can be observed. Adapted from McCarthy et al. (2011).

Kerogens are structurally complex large macromolecular structures over 1 kDa in size, consisting of aliphatic hydrocarbons with cyclic and aromatic compounds^8, 9^. It is classified into four types based on origin and degree of oxidation, assessed through oxygen-to-carbon and hydrogen-to-carbon ratios (Fig. 1). Kerogen type I, primarily derived from lacustrine algae, is rich in long-chain aliphatic hydrocarbons and highly reduced. Kerogen type II, formed from marine plankton, exhibits a more heterogeneous structure with both aliphatic and aromatic components. Kerogen type III, originating from terrestrial plants, is more oxidized, with a higher proportion of cyclic and aromatic compounds. Kerogen type IV consists of highly altered, residual organic material from older sediments, and is the most oxidized, displaying high oxidation and low hydrogen content^8, 10, 11^. Types I and II are predominantly found in shales, while types III and IV are more common in coals.

Thermal alteration of kerogen leads to the conversion of organic material into oil and natural gas^1^. Unlike other kerogen types, kerogen type IV lacks significant hydrocarbon potential and is therefore often overlooked^12^. However, kerogen type IV is of special interest in the extraterrestrial context. Non-biogenic organic compounds with structural and compositional similarities to kerogen type IV-like material are ubiquitous in the Universe, being found in the interstellar medium, on the surfaces of planets and moons, and in meteorites. Both kerogen type IV and extraterrestrial material share key compound classes, such as benzene, simple polycyclic aromatic hydrocarbons (PAHs), alkylated derivatives, and oxygen-, nitrogen-, and sulfur-containing aromatic compounds^12^. In addition, the oxidation and reworking steps that lead to the production of kerogen type IV, result in the removal of key biomarkers (e.g. alkanes and methyl phenols) which are present in the other kerogen types, but not in the extraterrestrial material^12, 13^. Consequently, kerogen type IV serves as a valuable analogue for the macromolecular material found in extraterrestrial settings and its microbial accessibility might give insights into its potential as a source of carbon to support indigenous life elsewhere or even as a future resource to support human exploration.

Kerogen is commonly viewed as recalcitrant to microbial degradation due to its macromolecular structure and low solubility^14, 15, 16^. However, studies indicate that certain microbial consortia can access and metabolize kerogen-derived compounds under specific conditions. Weathered, oxygenated black shales have been shown to support microbial growth^17, 18^, and biodegradation of coal and shale organic matter has been observed under anaerobic conditions^19, 20, 21^. Microbial activity may alter kerogen structure, releasing bioavailable carbon compounds that enter the carbon cycle. The degree of microbial accessibility likely varies with kerogen type, as oxidation state, aromaticity, and chemical composition influence bioavailability. For example, type I kerogen, with its long aliphatic chains, may be less accessible than more oxidized type III and IV kerogens, which contain functional groups that could facilitate microbial interactions^8^.

The bioremediation of coal and shale, such as through methanogenesis in syntrophic communities, has been extensively demonstrated (e.g. ^20, 21, 22, 23^). The deep subsurface biosphere harbors 12–20% of Earth’s total microbial biomass^24^, with microorganisms playing a central role in the transformation and mobilization of organic carbon. 80–99% of the carbon in sedimentary rocks such as coals and shales is stored as kerogen^25^. The remaining carbon consists of soluble organic carbon called bitumen, containing saturates, aromatics, resins and asphaltanes among others^25^. Microbial communities in these kerogen-rich, deep subsurface environments often rely on alternative electron acceptors, such as nitrate or sulfate, to drive anaerobic degradation of organic matter^19^. Anaerobic degradation of kerogen-rich shales has been linked to methanogenic and sulfate-reducing microbial consortia^25^. The kerogen-rich rocks containing types II and III, used in the present study, have previously been shown to enhance microbial growth in *Variovorax paradoxus* YC1^8^. The soluble organic carbon in kerogen-rich rocks could be biologically available for microbial degradation. In order to test the effect of the kerogens and rule out the effect of the bitumen, a few studies have focused on the anaerobic microbial degradation of isolated kerogen. The number of these studies is limited and these studies have focused on methanogenic consortia^25, 26^. The study by Vick et al. (2019) demonstrated microbial degradation of kerogen, but did not identify the specific type of kerogen in the coal. Meslé et al. (2015) demonstrated microbial degradation of kerogen types II and III. Despite these findings, a comprehensive understanding of how kerogen-rich rocks containing different kerogen types influence microbial growth and community structure remains absent.

This study aims to fill this knowledge gap, by addressing the question how the presence of kerogen-rich rocks, each containing one of the four kerogen types, influences microbial growth and community structure of an anaerobic community. In this paper, we investigated the growth and community structure of microorganisms grown in the presence of rocks each containing one of the four major kerogen types using the same initial microbial inoculum. We show how different growth patterns and communities are established in the different kerogen types. This provides an overarching insight into the interactions between these microorganisms and this deep subsurface carbon pool under controlled environmental conditions. The deep subsurface biosphere is shaped by chemical and environmental factors that can promote or inhibit microbial presence and growth. Organic content is particularly crucial for microorganisms, as carbon is an essential element for all life. Additionally, the deep subsurface encompasses a wide range of environmental variables, including pH, temperature, pressure, and fluid flow. To eliminate the confounding effects of these environmental differences, controlled laboratory experiments offer a way to isolate the influence of rock chemistry on the biosphere. Therefore, this study tests the effect of kerogen-rich rocks in set environmental conditions, such as room temperature and neutral pH values. An anaerobic microbial community was used that was adapted to growth on kerogen-like rich rocks. The effect of kerogen-rich rocks on the growth and composition of the microbial community was tested with colony-forming units (CFU) counts and 16S rRNA amplicon sequencing respectively. Gaseous metabolite production during the microbial growth was investigated using gas chromatography (GC. The surface of the kerogen-rich rocks and the microorganisms were visualized using scanning electron microscopy (SEM). By mimicking the natural environment under controlled conditions, this study narrows the focus to the chemical composition of kerogen-rich rocks, particularly their carbon content, bridging the gap between natural systems and laboratory experiments. This study significantly advances our understanding of the interactions between microorganisms and kerogen-rich rocks by demonstrating that kerogen-rich rocks support specific anaerobic microbial assemblages and promote microbial growth, potentially contributing to kerogen degradation.

## Results

### Microbial growth

**Fig. 2:**
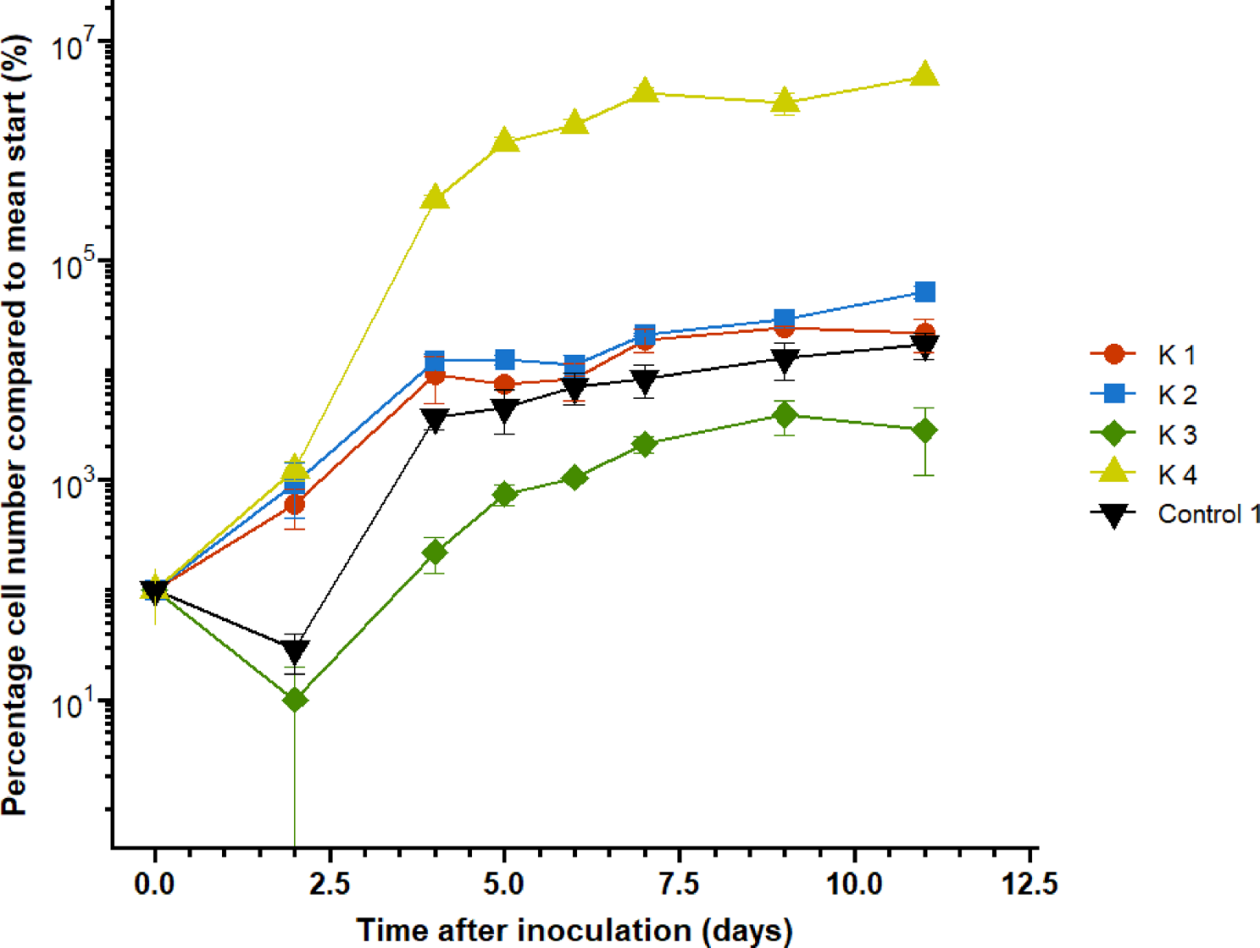
Microbial growth curves based on CFU counts in microcosms containing powdered rocks rich in kerogen type I, II, III and IV, as well as control 1 (containing no kerogen). CFU counts are compared to the mean CFU count at the start of the experiment, which is 100%. Data points represent the mean and error bars represent the standard error.

Microbial growth was indicated by CFU counts. An ANOVA and post hoc Tukey test showed that microbial growth was significantly enhanced by the presence of kerogen type IV-rich rock, while a trend showed microbial growth inhibition by the presence of kerogen type III-rich rock (Fig. 2) (ANOVA: *F*-statistic (4,10) = 7736; *p* value < 0.001). The microcosms contained 10^3^–10^5^ CFU/mL at the end of the experiment. Microbial growth was not significantly influenced by the presence of kerogen type I- and II-rich rocks. Non-biological controls showed no contamination in the kerogen-rich rocks containing kerogen type II-, III and IV-rich rocks (data not shown). Kerogen type I-rich rock however did show some contamination (data not shown).

A bacterial cell contains on average 2 * 10^–13^ g carbon^27^. The microbial community in control 1 (with medium but without kerogen) grew from 5.83 * 10^2^ CFU/mL to 1.19 * 10^5^ CFU/mL, resulting in a growth of 5.9 * 10^5^ CFU per microcosm. As some of the microbial population will likely not be culturable, and is therefore missed in CFU counts, this growth is likely to be an underestimation, and is therefore described as the minimal cell concentration. The CFU-observed growth requires 1.19 * 10^–7^ g carbon, while 0.0101 g carbon was present in a microcosm from the acetate. Therefore, only 1.18 * 10^–3^% of the available carbon was used by the microorganisms when a stationary phase was reached. This means that carbon was not a limiting factor in the growth experiment. Further, this indicates that acetate was still available to the microorganisms at the end of the growth curve in the conditions with kerogen-containing rocks. Thus, carbon was not a limiting factor in these conditions either.

### pH measurements

The pH of the microcosms at the start of the experiment was circumneutral (Table 1 and 2). An ANOVA (*F-*statistic (9, 18) = 454.7; *p *value < 0.001) and post hoc Tukey test showed that the pH of the kerogen type II-rich rock containing biological microcosms increased significantly during the experiment, while the pH of kerogen type IV-rich rock containing microcosms significantly decreased during the experiment (Table 2). This increase in the pH of the microcosms containing kerogen type II- and decrease in kerogen type IV-rich rocks was also observed in the non-biological samples (*F*-statistic (11,10) = 578.8; *p* value < 0.001) (Table 2). At the end of the experiment, kerogen type II-rich rock containing microcosms had a significantly higher pH and kerogen type IV-rich rock containing microcosms had a significantly lower one than all other conditions. In addition, kerogen type I-rich rock-containing microcosms had a significantly higher pH than control 1 microcosms. The other kerogen-containing microcosms did not differ significantly from control 1.

**Table 1:** pH biological samples.

Sample name	Starting pH	Final pH
Control 1	7.05	7.04 ± 0.02
Kerogen type I	7.15 ± 0.02	7.20 ± 0.03
Kerogen type II	7.09 ± 0.01	7.63 ± 0.02
Kerogen type III	7.14 ± 0.02	7.11 ± 0.02
Kerogen type IV	6.80 ± 0.04	5.98 ± 0.08

pH (average ± standard deviation) before inoculation (starting) and after 11 days of microbial growth (final). All samples were taken in triplicate, except from the starting pH of control 1, which was measured once.

**Table 2:** pH non-biological samples.

Sample name	Starting pH	Final pH
Control 1	7.19 ± 0.06	7.12 ± 0.04
Kerogen type I	7.21 ± 0.02	7.12 ± 0.04
Kerogen type II	7.11 ± 0.01	7.58 ± 0.00
Kerogen type III	7.19	7.12
Kerogen type IV	7.00 ± 0.01	5.31 ± 0.01

pH (average ± standard deviation) at the start and after 11 days (final). All samples were taken in duplicate, except for kerogen type III, which was measured once.

### 16S rRNA Gene Amplicon Sequencing

The raw OTU feature table has 2,742 OTUs. The rarefied OTU table has 2,504 OTUs and a sampling depth of 33,722 sequences with a quality score of ≥ 20. The negative control and negative PCR control contained 4,515 and 1,542 sequences respectively divided in 187 OTUs.

**Fig. 3:**
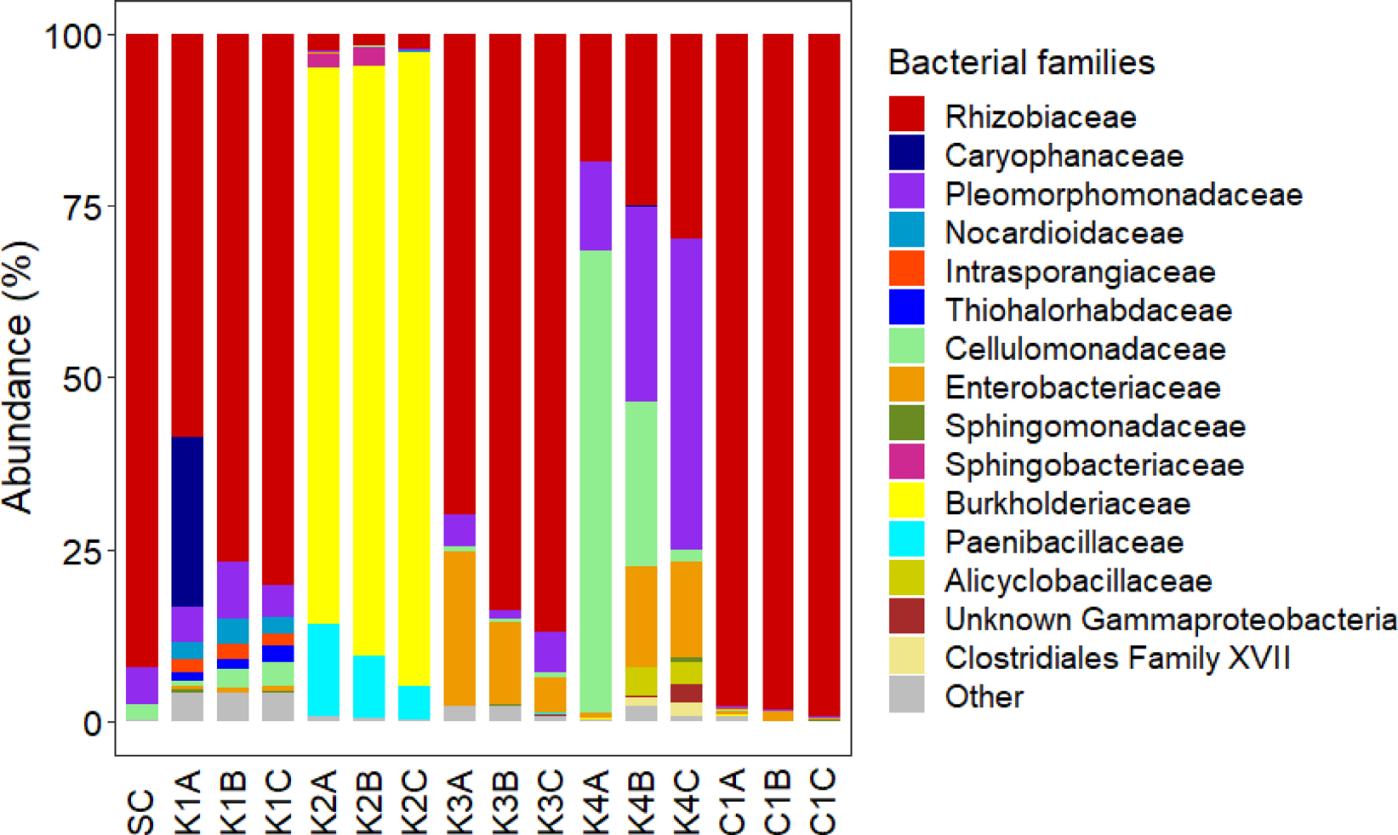
Microbial communities growing in the presence of kerogen-rich rock. Relative abundance of microbial communities in batch cultures at the family taxonomic level. Composition of starting culture (SC) and communities in the presence of kerogen type I-rich rock (K1A-K1C), type II-rich rock (K2A-K2C), type III-rich rock (K3A-K3C), type IV-rich rock (K4A-K4C) and without kerogen-rich rock: Control 1 (C1A-C1C). Samples A, B and C are biological replicates. ‘‘Other’’ contains family-level taxa at an abundance smaller than 1%.

**Fig. 4:**
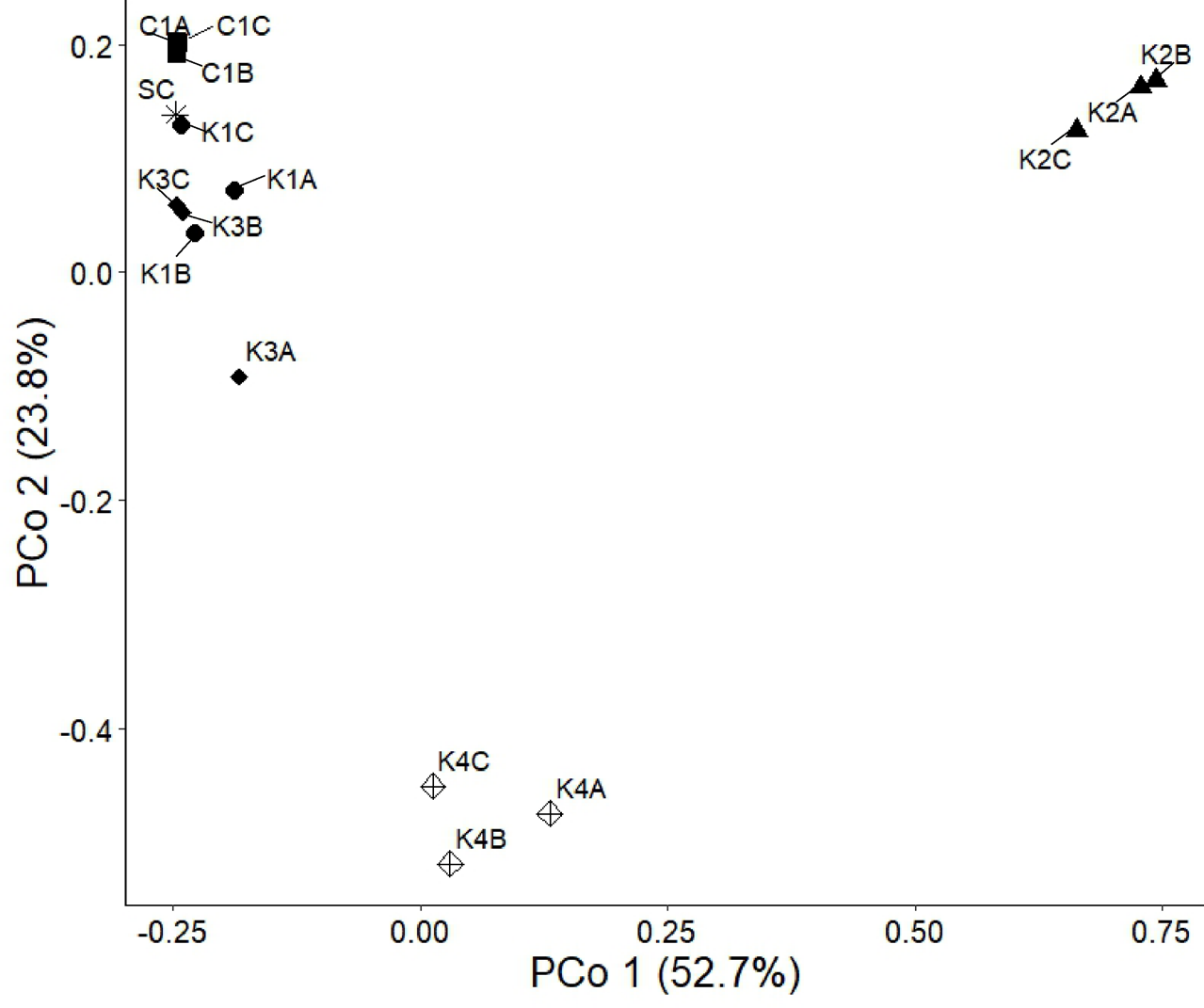
Principal Coordinate Analysis (PCoA) plot of microbial communities. PCoA plot of microbial community compositions at the bacterial family level based on Fig. 3. Samples: the starting culture (SC) and communities in the presence of kerogen type I-rich rock (K1A-K1C), type II-rich rock (K2A-K2C), type III-rich rock (K3A-K3C), type IV-rich rock (K4A-K4C) and without kerogen-rich rock: Control 1 (C1A-C1C). Samples A, B and C are biological replicates.

The microbial community composition of the communities at the start and end of the second experiment is shown in Fig. 3. The starting culture (SC) had 92% of sequences assigned to the bacterial family Rhizobiaceae and contained a total of five bacterial family-level taxa. The communities after growth in the presence of kerogen type II-rich rock (K2A-K2C) and type IV-rich rock (K4A-K4C) were the most different from the starting culture, which was confirmed with a principal coordinate analysis (PCoA) (Fig. 4).

The communities with kerogen type II-rich rock mainly contained Burkholderiaceae (81, 86 and 92%), some Paenibacillaceae (14, 9.1 and 4.9%), and just 2.5, 1.5 and 2.2% Rhizobiaceae, respectively. In samples K2A and K2B, one OTU (AB487328.1.1370) was responsible for most of the Burkholderiaceae present (resp. 79 and 82%), while the Burkholderiaceae of K2C were slightly more diverse, with three OTUs (AB487328.1.1370, JX222290.1.1490 and KC541176.1.1524) containing resp. 33, 27 and 20%.

In the presence of kerogen type IV-rich rock, the community shifted to containing mainly Cellulomonadaceae (67, 24 and 1.9%), Pleomorphomonadaceae (13, 29 and 45%) and Enterobacteriaceae (0.8, 15 and 14%), while still containing a high abundance (19, 25 and 30%) of Rhizobiaceae. In K4A and K4B, one OTU (MLJW01000851.1934.3445) was responsible for the majority of the Cellulomonadaceae present (resp. 67% and 22% of the total population). One OTU (JQ659759.1.1352) was responsible for the majority of the Pleomorphomonadaceae in all kerogen type IV-rich rock samples (resp. 10, 22 and 33%). The community in the presence of kerogen type I- and III-rich rock was altered compared to the inoculum, but the majority of these organisms was Rhizobiaceae, the same as in the starting culture. The control 1 communities changed the least from the inoculum. The Rhizobiaceae in the starting culture was dominated by two OTUs (AF529121.1.1399 and DQ096643.1.1404), respectively 59 and 29% of the total population. These were also the most abundant Rhizobiaceae in all K1, K3 and C1 samples.

**Fig. 5:**
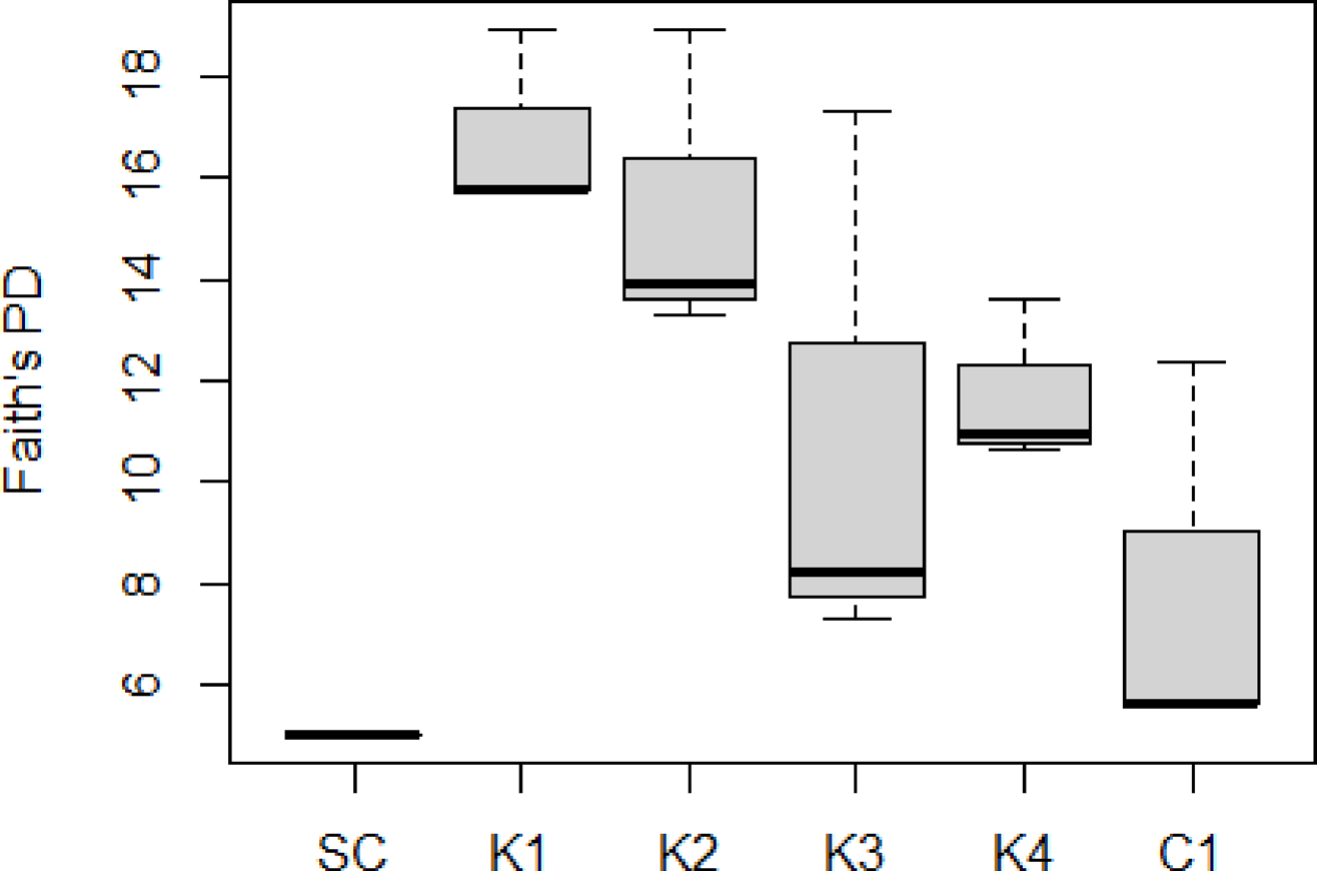
Phylogenetic diversity of microbial communities. Faith’s phylogenetic diversity (PD) of the starting culture (SC) and in triplicate microcosms containing kerogen type I-rich rock (K1), type II-rich rock (K2), type III-rich rock (K3), type IV-rich rock (K4) and Control 1 (C1).

Faith’s phylogenetic diversity (PD) showed that the starting culture had a lower alpha diversity than all other conditions (Fig. 5). The Faith’s PD did differ significantly from one another (ANOVA: *F-*statistic (5,10) = 3.377; *p *value = 0.048), but no significant pairwise comparisons were found using a post hoc Tukey test.

### Gaseous metabolite production

**Fig. 6:**
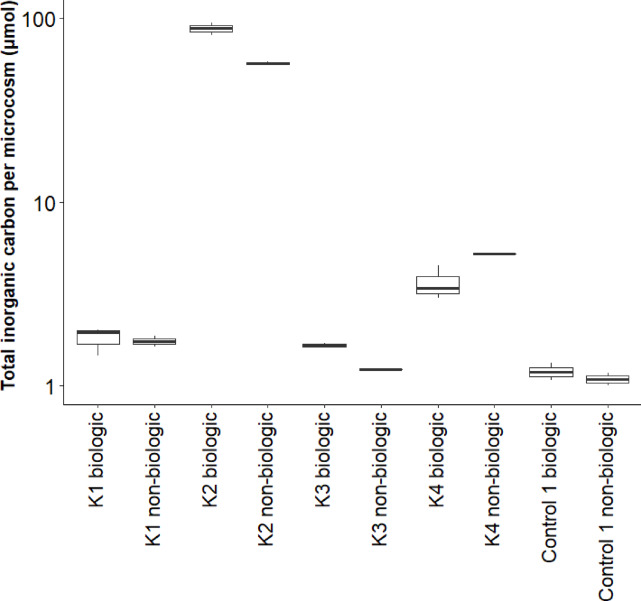
Total inorganic carbon (TIC) in µmol per microcosm after 11 days of incubation, both in the biological and non-biological experiment. K2 samples were significantly higher than all other samples. K2 biological was significantly higher than K2 non-biological. No other significant differences were observed. Boxes in the box plots show the median, upper and lower quartile. Error bars depict the smallest and largest values.

Total inorganic carbon (TIC) production was calculated from the measured CO_2_ concentration using Henry’s Law and carbonate equilibria equations (Supplemental Equations). The TIC in both biological (by the presence of rock powder, water and the microbial community in the microcosms) and non-biological (by the presence of rock powder and water in the microcosms) samples is shown in Fig. 6. An ANOVA and post hoc Tukey test showed that the biological samples of kerogen type II-rich rock contained significantly more TIC than all other biological samples and that the non-biological samples of kerogen type II-rich rock contained significantly more TIC than all other non-biological samples (ANOVA: *F-*statistic (9,14) = 296.9; *p *value < 0.001). In addition, samples of kerogen type II-rich rock contained significantly more TIC in the biological samples than in the non-biological samples (ANOVA: *F-*statistic (9,14) = 296.9; *p *value < 0.001). Other conditions did not differ significantly from each other, and no significant TIC production was observed in any of the other samples. No carbon dioxide was observed in the anaerobic chamber in which the experiments were conducted. Methane and higher alkane production were not observed.

**Fig. 7:**
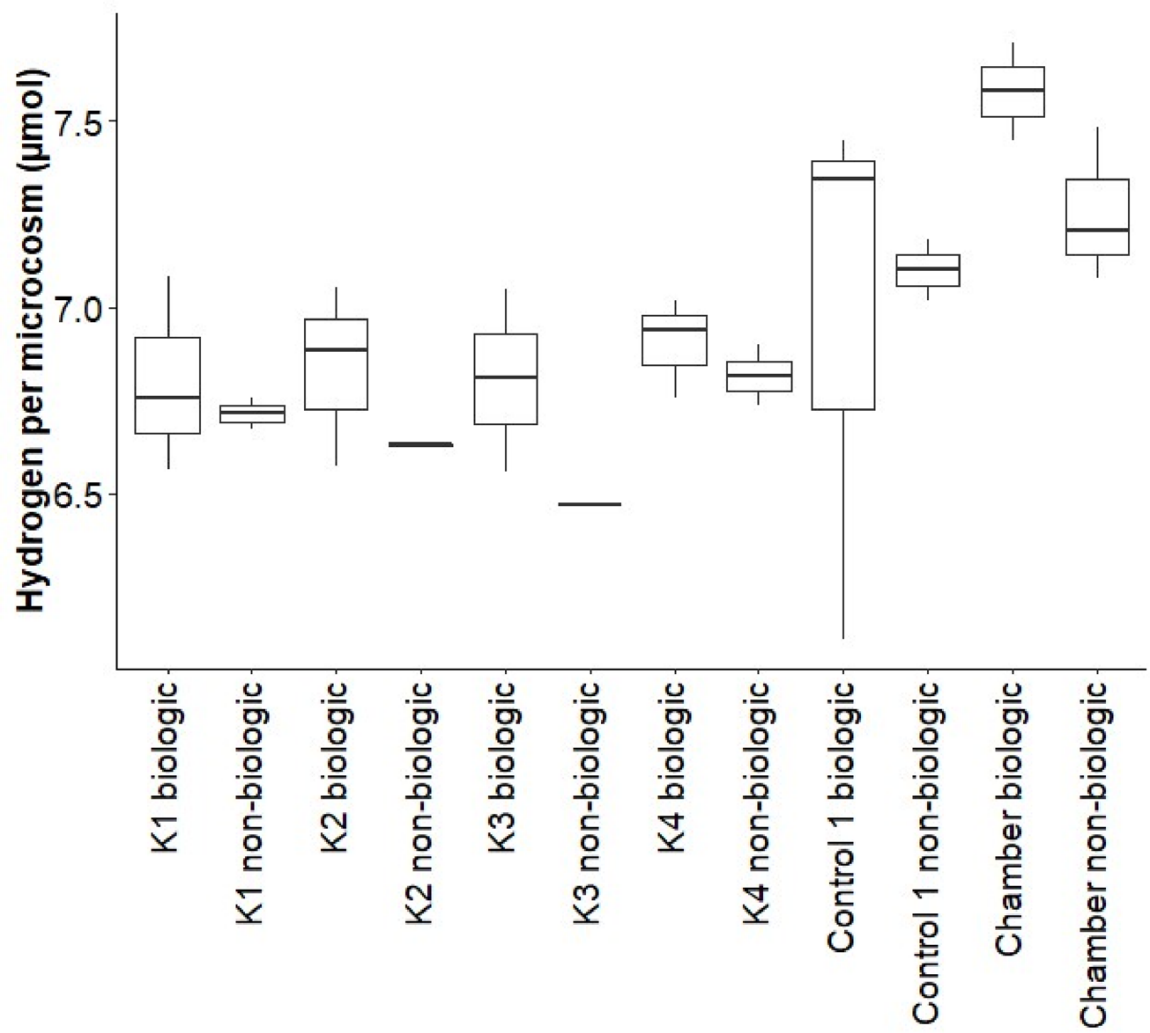
Hydrogen in µmol per microcosm after 11 days of incubation, both in the biological and non-biological experiment; as well as the hydrogen present in the anaerobic chamber in the same volume of gas at the end of the experiments.

Hydrogen gas was present in the experimental setup at an average of 2.04% in the anaerobic chamber during the biological experiment and an average of 1.96% during the non-biological experiment. This corresponds to 7.58 and 7.26 µmol per volume of headspace in a microcosm (Fig. 7). H_2_ was neither produced nor consumed in the microcosms, with no significant difference in hydrogen concentration in the microcosms at the end of the experiments compared to the anaerobic chamber (ANOVA: *F-*statistic (11,17) = 1.719; *p *value = 0.153). A significant deviation in H_2_ concentration in Control 1 biotic was observed, which was caused by one outlier with a low H_2_ concentration. This could for example be caused by gas leakage out of the microcosm.

### Scanning electron microscopy

**Fig. 8:**
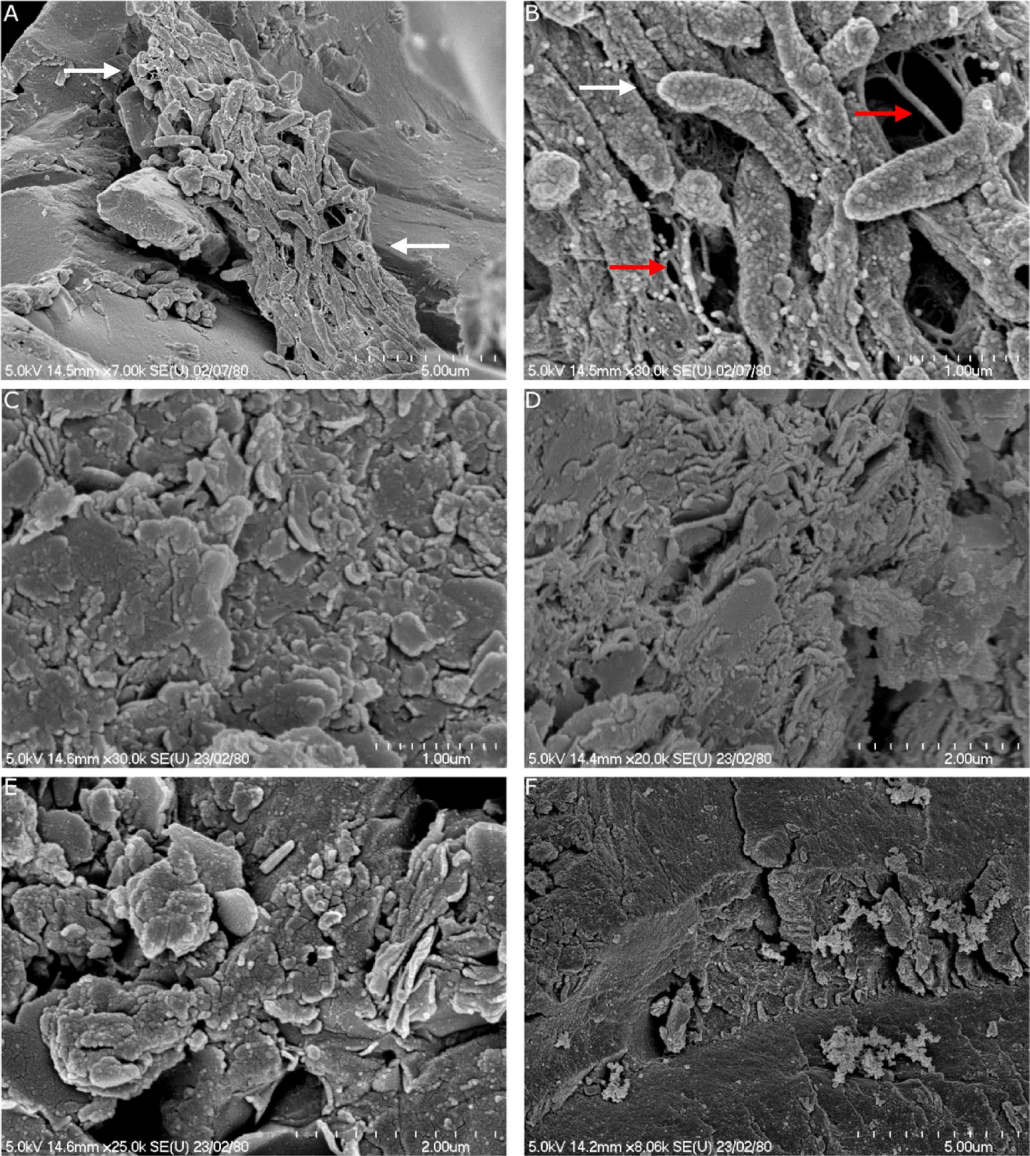
Scanning electron microscopy (SEM) images of kerogen-rich rocks and bacterial communities embedded in the rocks. (**A**) A group of cells (indicated with white arrows) on kerogen type III-rich rock. Scale bar is 5 µm. (**B**) The surface of cells (white arrows) and filamentous excretion (red arrows) on kerogen type III-rich rock. Scale bar is 1 µm. (**C**) Surface of kerogen type I-rich rock. Scale bar is 1 µm. (**D**) Surface of kerogen type II-rich rock. Scale bar is 2 µm. (**E**) Surface of kerogen type III-rich rock. Scale bar is 2 µm. (**F**) Surface of kerogen type IV-rich rock. Scale bar is 5 µm.

Using Scanning Electron Microscopy (SEM), microbial colonies were observed on the kerogen type III-rich rock (Fig. 8A,B). The rod-shaped bacteria were clustered together and covered in spherical excretions (Fig. 8B). Filamentous excretions between the bacteria were also observed (Fig. 8B). The surface of all kerogen-rich rocks shows small, rod- and sphere-like structures (Fig. 8C–F). However, kerogen type IV-rich rock contains fewer of the rod- and sphere-like structures than the other rocks and the material appeared to have a more laminated sheet-like structure (Fig. 8F).

## Discussion

This study provides valuable insights into the interactions between microorganisms and kerogen-rich rocks, each containing one of the four kerogen types. This highlights the significant variations in microbial growth and community composition based on the type of rock. The findings underscore the importance of the composition of the kerogen-rich rock in shaping microbial communities and influencing microbial degradation processes.

This study effectively minimized environmental differences between kerogen-rich rocks, while maintaining the natural occurrence of the rock. This was obtained by investigating complete rock samples including the rock matrix in a controlled environment. Therefore, this study links the natural environment to laboratory experiments that use extracted kerogen, and shows the influence of the complete kerogen-rich rock—including further differences in the matrix—on a microbial community.

### Kerogen type-specific microbial interactions

The results of this study confirm that kerogen-rich rocks exert distinct effects on microbial communities, depending on the kerogen type. Kerogen type I- and II-rich rocks showed no significant effects on microbial growth. The absence of microbial growth enhancement in these conditions could indicate that the organic material in these rocks is either not bioavailable or too recalcitrant for microbial degradation. The presence of kerogen type III-rich rocks inhibits microbial growth. Next to the differences in the composition of the soluble organic carbon and the matrix of the rock compared to the other investigated rocks, the composition of kerogen type III could explain this inhibition. Kerogen type III is abundant in phenolic compounds, which could explain this toxicity^12^, since phenolic compounds are highly toxic, resistant to biological degradation and can accumulate in organisms^28^. Kerogen type IV-rich rocks, by contrast, significantly enhanced microbial growth, which aligns with previous findings that kerogen type IV-like material in carbonaceous chondrites can support microbial life^29^. Kerogen type IV consists of reworked and oxidized material, which results in the presence of benzene, simple polycyclic aromatic hydrocarbons (PAHs), alkylated derivatives, and oxygen-, nitrogen-, and sulfur-containing aromatic compounds^12^. These can potentially be more easily degraded by microorganisms than the phenol-rich composition of kerogen type III. The increased growth could also be related to the fact that the communities in this study were grown and subcultured from a culture grown on a carbonaceous chondrite. This would demonstrate the resemblance between kerogen type IV-rich rock and carbonaceous chondrites. This increased growth points to the bioavailability of carbon and possibly other nutrients from kerogen type IV, as well as other differences between the rock types, such as the soluble organic carbon and the matrix, providing a favorable environment for microbial proliferation. However, the experiment’s reliance on CFU counts as a sole method of estimating growth presents certain limitations. The CFU method is known to favor the growth of certain taxa while potentially underestimating others^30^. Future studies could employ non-cultivation-based approaches, such as qPCR, to gain a more comprehensive understanding of microbial growth dynamics and community composition.

### Microbial community composition and adaptation

Changes in the microbial community composition, particularly the increase in Burkholderiaceae in the presence of kerogen type II-rich rock, and Cellulomonadaceae and Pleomorphomonadaceae in the presence of kerogen type IV-rich rock, suggest that certain taxa are better adapted to specific kerogen-rich environments.

The Burkholderiaceae family is extremely diverse and includes both facultatively anaerobic and strictly aerobic organotrophs, in addition to facultative and obligate lithotrophs^31^. Burkholderiaceae species can oxidize acetate to CO_2_^32^, which could explain the CO_2_ production in the biological kerogen type II microcosms. Additionally, Paenibacillaceae have also increased in abundancy in the presence of kerogen type II-rich rocks, which could have produced the CO_2_. Cellulomonadaceae are aerobic to facultatively anaerobic bacteria, that can decompose a wide variety of macromolecules, e.g. cellulose, starch, xanthan, chitin, DNA and gelatine^33^. Several kinds of acid, such as acetic acid, and CO_2_ can be produced from these metabolisms. This ability could be beneficial for the potential kerogen degradation. The family Pleomorphomonadaceae is a recently reported family^34^ of mainly aerobic, diazotrophic members. More research on this family is needed to understand which characteristics of this family allow the growth in the presence of kerogen type IV-rich rock.

The adaptive behavior of these three bacterial families could indicate the presence of specialized pathways for kerogen or bitumen degradation, or the ability to utilize secondary metabolites released from the kerogen matrix. However, the observation that the community adapted to kerogen type II-rich rock without significant CFU-observed growth enhancement raises questions about the underlying mechanisms. Are the community metabolizing compounds released from the kerogen, or is it merely adapting to a changed environment? A deeper biochemical analysis of the products released from kerogen during microbial interactions could provide more insight into these processes.

The shifts in community diversity, as indicated by Faith’s phylogenetic diversity index, suggest that different kerogen types offer a broader range of substrates than the acetate-based medium alone. Therefore, families that were present at a very low abundance in the starting culture and thus not detected by 16S sequencing could increase in abundancy and get detected in the kerogen-containing cultures. This supports the idea that microbial communities are more diverse in environments with varied organic matter.

SEM images show rock-assembled microbial cells that are grouped together and embedded in kerogen type III-rich rock. Many of the bacterial families present in the kerogen type III-rich condition are rod-shaped like the bacteria on the SEM images, such as Rhizobiaceae, Enterobacteriaceae and Pleomorphomonadaceae. However, no flagella were observed, which all Rhizobiaceae species are known to possess^35^. The filamentous excretions between the microorganisms are likely to be extracellular polymeric substrate (EPS). EPS provide support and protection of microbial communities in harsh environments^36^. Spherical ‘excretions’ were also observed. These could be of various origins, such as small fragments of the rock, or a cellular response to the new environment. Next to the excretion of filaments, clustering together of cells and the encrustation of cell walls with a rough surface has been observed before in harsh environments^37^. These features are thought to be stress responses which protect the cells from the harsh environmental conditions^37^. Since the kerogen type III-containing condition inhibits microbial growth, it can be expected that this environment is a harsh, stressful environment for the microbial community to which the microbial cells will execute stress responses.

The SEM study did not discover microbial cells in any of the other kerogen-containing samples. This was likely to be caused by the low biomass concentration of the samples. Therefore, the SEM study only provides limited information regarding the microbial growth.

CO_2_ production was observed both non-biologically and biologically in the presence of the kerogen type II-rich rock by an increase in the total inorganic carbon. Non-biologic CO_2_ production can be caused by the dissolution of adsorbed CO_2_ from the kerogen-rich rock in water^38^. Biological CO_2_ production was only observed in the presence of the kerogen type II-rich rock, which could be produced by Burkholderiaceae and Paenibacillaceae, two bacterial family-level taxa that are highly abundant in kerogen type II, but not in the other conditions. Burkholderiaceae species are known to oxidize acetate to CO_2_^32^, and Paenibacillaceae are chemoorganoheterotrophs that can produce CO_2_ as well^39^. The CO_2_ could either be produced from the acetate that was present in the medium, or from the degradation of kerogen. The lack of observation of CO_2_ production in the other conditions, as well as the lack of observation of H_2_ and alkane production across all conditions, could be caused by a lack of metabolite production, but also by detection limitation of the equipment. The microcosms contained low cell numbers at the end of the experiment (10^3^–10^5^ CFU/mL), making it plausible that gaseous metabolite production would remain below the detection limit. However, if the lack of metabolite observation was caused by the lack of metabolite production of these gaseous metabolite, this would support the statement that these kerogen-rich rocks are recalcitrant to microbial degradation into alkanes, carbon dioxide and hydrogen.

### Implications for extraterrestrial habitability

The findings of this study also have broader implications for the potential habitability of extraterrestrial environments. Kerogen type IV, which is chemically similar to macromolecular material found on the Martian surface and in carbonaceous chondrites^12, 13^, could potentially serve as a carbon or energy source for extraterrestrial microorganisms. Waajen et al. (2022, 2024) found that bacteria can use the carbon in carbonaceous chondrites as their sole carbon source. This is in line with the growth-enhancing effect of kerogen type IV-rich rocks, which suggests that similar material on Mars or other celestial bodies could support microbial life, particularly if conditions allow for the presence of liquid water. As 70% of the organics in carbonaceous chondrites are insoluble, kerogen-like macromolecular material^40, 41, 42^, this indicates that these organics increase the habitability of the environments in which these carbonaceous chondrites landed. However, while the analogy to Martian conditions is compelling, it is crucial to recognize the differences in environmental context as well as differences between terrestrial kerogens with a biological source and extraterrestrial kerogen-like organics with an abiotic source. For instance, the role of radiation, atmospheric composition, and temperature must be considered when evaluating the potential for microbial life in extraterrestrial environments.

## Conclusion

This study underscores the complexity of microbial interactions with kerogen-rich rocks, highlighting the specific influences of the composition of the kerogen-rich rocks on microbial growth and community composition. The results emphasize that differences in the soluble organic carbon, the matrix of the rock or the structure of kerogen can play a key role in microbial degradation processes and may influence the habitability of subsurface environments. Different types of kerogen-rich rocks were found to affect microbial community growth and composition in distinct ways, suggesting that these rocks play a significant role in shaping the microbial biosphere.

Carbon mobilization from kerogen-rich rocks would have large implications for our understanding on the availability of carbon in the largely unexplored deep subsurface. Whereas the deep subsurface is thought to contain many carbon-limited environments^43^, the mobilization of deep subsurface carbon previously thought to be recalcitrant, would imply that this may not be the case. This has large implications for our understanding of the deep biosphere ecology. While this study focused on a single microbial community, the data reveal that different kerogen-rich rocks are mobilized to varying extents by microorganisms, and that their structure is a key determinant in microbial activity.

Beyond deep subsurface ecology, these findings have broader astrobiological implications. The ability of microorganisms to interact with and potentially utilize kerogen-like materials suggests that similar organic substrates on other planets could contribute to the habitability of such environments. Identifying biosignatures associated with kerogen degradation, such as specific metabolic byproducts or microbial community structures, could guide future astrobiological investigations, particularly in the search for life on Mars and other celestial bodies.

Future research should expand on these insights by exploring microbial responses across diverse environmental conditions, community compositions, and kerogen substrates. Further experiments are needed to investigate the degradation of kerogens and the soluble organic carbon in these rocks, and the metabolic pathways involved in these processes. Moreover, by investigating diverse environmental conditions, it should be assessed how these processes might vary across planetary environments. By deepening our understanding of microbial-deep subsurface organic interactions, we can refine our understanding for the broader potential for life in subsurface and extraterrestrial environments, model the deep biosphere ecology and enhance our search for extraterrestrial life.

## Materials and methods

### Kerogens

Four kerogen-rich rocks were used, which were provided by collaborators at Imperial College London (Table 3). The vitrinite reflectance value (VRo) is a maturity indicator that indicates the level of thermal metamorphism (maturity) experienced by the samples^44^. The indicator ranges from the start (0.6) to the middle (0.9) of the ‘oil window’, the range in which oil can be generated from organic-rich rocks (Fig. 1)^44^. Since kerogen type IV does not have the potential to generate oil or gas, this does not have a VRo value. The thermal maturity of kerogen type IV rock has been reported by the hydrogen index (17) and oxygen index (127) using Rock–Eval pyrolysis^45^. By using pyrolysis–gas chromatography-mass spectrometry (Py-GC–MS), Montgomery et al. (2016) showed that kerogen type I and II of the samples used in the present study are rich in straight-chain hydrocarbons, while type IV is rich in cross-linked aromatic units. GC–MS on an organic solvent extract of the type III sample showed that it contains a range of alkylated aromatic, phenolic and oxygen-containing aromatic compounds^46^.

**Table 3:** Sample origin.

Kerogen type	Rock type	Location	Age	TOC (%)	VRo (%)	References
Type I	Lacustrine shale	Port Edgar, West Lothian, UK	Carboniferous (359–299 Mya)	13.43	0.9	^44, 47^
Type II	Marine shale	Monmouth Beach, Dorset, UK	Jurassic (201–145 Mya)	8.14	0.6	^44, 48^
Type III	High volatile bituminous coal	Daw Mill, Warwickshire, UK	Carboniferous (359–299 Mya)	56.4	0.6	^8, 46^
Type IV	Charcoal	Wealden Beds, Durdle Door, Dorset, UK	Cretaceous (145–66 Mya)	53.46	–*	^44, 45^

Details of kerogen-rich rock samples: rock type, location, age, total organic carbon (TOC) and vitrinite reflectance value (VRo). *Although kerogen type IV does not have a VRo value, the hydrogen index and oxygen index by Rock–Eval pyrolysis are known (respectively 17 and 127)^45^.

### Microorganisms

An anaerobic microbial community was used that was sampled from the anoxic benthic subsurface of a freshwater pond in Edinburgh, Scotland (55°55′31.3"N, 3°11′45.8"W). This pond was eutrophic and contained a variety of complex organics^49, 50, 51^. This environment was chosen since it is anoxic and the presence of the variety of organics allowed for the presence of a wide variety of organic-degrading pathways. After sampling, the microorganisms were grown anaerobically with a carbonaceous chondrite, a meteorite in which 70% of the organics are kerogen type IV-like organics, as the sole carbon and energy source. Microbial growth was carried out by three consecutive anaerobic microcosms containing 50 µL inoculum, 5 mL molecular grade water and 0.5 g powdered carbonaceous chondrite for 2 months each^27^. Details are described earlier^27^.

### Preparation of anaerobic cultures

Glassware was made organic-free and butyl-rubber stoppers were cleaned as described earlier^27, 52^. The kerogen-rich rocks were powdered with a mortar and pestle that had been heated to at least 500 °C for a minimum of 5 h in a Carbolite 1100 °C Chamber furnace to purge the mortar and pestle of organics and sterilise them. Inside an anaerobic chamber (Coy Laboratory Products) containing 98% N_2_ and 2% H_2_ at 1 atm, anoxic microcosms were made in 12.5 mL glass bottles containing 5 mL liquid M9 medium (3 g/L KH_2_PO_4_; 7 g/L Na_2_HPO_4_; 1 g/L NH_4_Cl; 0.5 g/L NaCl; 0.12 g/L MgSO_4_; 0.011 g/L CaCl_2_) with 6.94 g/L sodium acetate (CH_3_COONa). We added acetate to provide a background carbon source. The reason for this was to be able to distinguish between whether, if we observed a lack of growth by the community, it could be caused either by inhibition by the kerogen-containing rocks, or by a lack of ability to use the kerogens as an organic supply. The addition of acetate allowed us to investigate whether the kerogen-containing rocks could augment growth with a background carbon source and whether the kerogen-containing rocks would change the microbial community composition compared to a community growing in acetate alone as a carbon source. The amount of acetate was chosen to resemble the amount of carbon that was present in the microcosms with the carbonaceous chondrites described above. This high acetate concentration could have shaped the microbial community prior to the start of the experiment, but testing had shown that the overall microbial community grew well in this medium (data not shown). In addition, each microcosm contained 0.5 g of either one of the powdered four kerogen-rich rocks as described above, or no rocks for control 1. The microcosms are named as follows: Microcosms in the presence of kerogen type I-rich rock (K1A-K1C), type II-rich rock (K2A-K2C), type III-rich rock (K3A-K3C), type IV-rich rock (K4A-K4C) and without kerogen-rich rock: Control 1 (C1A-C1C). Samples A, B and C are biological replicates. Non-biological controls were performed with M9 medium, sodium acetate and rock powder, but without inoculum, to investigate potential contamination of the kerogen-rich rocks. The pH of each microcosm was adjusted to 7 prior to inoculation using a Jenway 3510 pH meter (Cole-Parmer, Staffordshire, UK) and InLab Semi-Micro-L pH electrode (Mettler Toledo Ltd, Leicester, UK). The community as described above was grown anaerobically in microcosms containing 5 mL M9 medium with sodium acetate and transferred three times at late exponential phase to establish a stable community on this medium. The third microcosm was used as the inoculum of the growth experiment and is referred to as the starting culture (SC).

### Microbial growth

The first experiment was conducted by growing the microbial community anaerobically for 11 days in triplicate batch cultures at room temperature. Growth of the community was measured by colony-forming units (CFU) counts on LB agar plates (according to DSM 381 [DSMZ; Deutsche Sammlung von Mikroorganismen und Zellkulturen]: 10.0 g/L tryptone; 5.0 g/L yeast extract; 10.0 g/L NaCl; 20 g/L Agar; pH 7.0). Plates were incubated anaerobically at room temperature. CFU counts were obtained by single plate-serial dilution spotting (SP-SDS) of the communities multiple times throughout the experiment^52^. Final cell concentrations of the different conditions were compared using an ANOVA and a post hoc Tukey test.

We attempted direct cell counting of the cell abundance. However, non-specific binding of DNA-staining dye to kerogen-containing rock fragments made this approach highly unreliable and it was not possible to reliably separate the cells from the rock material to gain reliable quantitative data. We therefore chose to examine cell abundance by CFU abundance.

pH was measured in triplicate at the end of the growth experiment using a Jenway 3510 pH meter (Cole-Parmer, Staffordshire, UK) and InLab Semi-Micro-L pH electrode (Mettler Toledo Ltd, Leicester, UK). An ANOVA and post hoc Tukey test were performed to compare the pH of the microcosms prior to inoculation to the end of the experiment.

Non-biological controls were incubated anaerobically in duplicate for 11 days at room temperature. Due to a processing error, one of the non-biological control samples containing kerogen type III-rich rock could not be analysed. Microbial contamination was investigated by CFU counts using SP-SDS on LB agar plates at the start and end of the experiment. The pH of the microcosms was measured at the start and end of the experiment.

### 16S rRNA gene amplicon sequencing and analysis

In the second experiment, the microbial community was grown in triplicate microcosms for 11 days on M9 medium with sodium acetate and one of the powdered kerogen-rich rocks. A control (Control 1) was included which contained medium but no kerogen-containing rock. DNA was extracted from the microcosm containing the microbial community at the start of the growth experiment using the DNeasy PowerLyzer PowerSoil Kit (QIAGEN GmbH, Germany) according to the manufacturer’s instructions. Additionally, DNA extraction was performed on a negative control, containing no microcosm sample, to test for the presence of any DNA in the extraction kit. DNA concentration was measured using a Qubit 3 Fluorometer (Invitrogen, USA), and samples were stored at -20 ºC before shipment for library preparation and 16S rRNA amplicon sequencing.

Libraries were prepared according to the manufacturer’s instructions with minor modifications. The primers 341F (CCTACGGGNGGCWGCAG) and 805R (GACTACHVGGGTATCTAATCC) were used for V3-V4 region amplification of the 16S rRNA gene^53, 54^. Five microliters of extracted DNA were used for the first round of PCR to amplify the V3-V4 region (10 µL was used for samples K1B, K3A, K3B, K3C, K4B, K4C due to low DNA concentration) with Platinum *Taq* DNA Polymerase High Fidelity (Invitrogen, USA). In addition to the community containing samples and the negative control sample, a negative PCR control sample with no DNA template was included. The following thermal profile was used for the first round of PCR: 95 °C for 3 min, then 35 cycles of 95 °C for 30 s, 55 °C for 30 s and 68 °C for 30 s, followed by an extension of 68 °C for 5 min. Purification of the amplicons was performed using Mag-Bind Total Pure NGS beads (Omega Bio-tek). Using Nextera XT v2 indexes (Illumina), the second round of PCR was performed to attach dual indices and Illumina sequencing adapters. This round of PCR used the same thermal profile as the first round, but used 8 cycles instead of 35. DNA concentration of indexed libraries was measured using the Quant-iT PicoGreen dsDNA Assay Kit (Invitrogen), after which the libraries were normalized and pooled. The average size of the pooled library was determined using a Bioanalyzer High Sensitivity DNA chip (Agilent). Libraries were then sequenced using an Illumina MiSeq (Illumina, USA) to generate paired 300-bp reads.

FASTQ files (available according to the Data Availability statement) were analysed using the EDGE Bioinformatics Web-based platform^55^, which used QIIME2 version 2019.10.0 automated scripts^56^. The selected quality control method was operational taxonomic units (OTUs), with 97% similarity for binned OTUs. All sequences with a Q-score of at least 20 were retained. The minimum fraction of consecutive high-quality base calls to retain a read was 0.75, with a maximum of one ambiguous nucleotide per sequence. Demultiplexing and joining of the sequences were performed using QIIME vsearch methods and commands^57^. Retained sequences were binned with the qiime vsearch cluster-features-open-reference command and the SILVA-132–99 database. UCHIME (qiime vsearch uchime-ref command) removed chimeric sequences. The q2-feature-classifier plugin determined the taxonomic identity of the OTUs^58^. The MAFFT alignment tool^59^ and FastTree method^60^ were used with the qiime phylogeny align-to-tree-mafft-fasttree command in order to create a phylogenetic tree from the raw OTU feature table.

An abundance bar plot was created using the rarefied OTU table, of which all bacterial family-level taxa containing less than 10 sequences in all environmental conditions were excluded. OTUs that were present both in the negative control and the negative PCR control with more than 10 sequences were excluded as well. A Bray Curtis distance matrix was generated in QIIME2 (qiime diversity core-metrics-phylogenetic command) and was analysed using a principal coordinates analysis (PCoA).

Alpha diversity was analysed using the Qiime diversity core-metrics-phylogenetic command, which was applied to the rarefied OTU table. This generated the species diversity index Faith’s phylogenetic diversity (PD) for each sample. These were compared for the different conditions using an ANOVA and post hoc Tukey test.

### Gaseous metabolite production

Metabolite production was examined in the second experiment. Pre-evacuated 3 mL exetainers (Labco, UK) were filled with 3 mL gas from the headspace of the microcosms and controls at the end of the experiment. In addition, the atmosphere of the anaerobic chamber at the start of the experiment, when the kerogen-rich rocks powder was added to the microcosms. This was done using a syringe with needle to transfer the gas. Exetainers were stored at 4 °C until all samples were shipped to Newcastle University for gas analysis.

Hydrogen and carbon dioxide concentrations were analysed using a Thermo Scientific Trace Gas Chromatograph with a helium-pulsed discharge detector (PDD) with a 10 times attenuated detector sensitivity. From the exetainers, 150 µl of gas was sampled using a 250 µl SRI gastight valved syringe. Prior to injection, the volume was reduced to 100 µl to over-pressurise the sample, after which the valve opened before injection to flush residual air from the needle and allow the sample pressure to equilibrate to 1 atmosphere. The 100 µl was then injected onto a 2m micropacked Shin Carbon ST 100/120 mesh column (1/16-inch OD, 1.0 mm ID column), with a constant flow (10 mL min^-1^) of helium carrier gas. The column temperature was 60 °C, the injector and detector temperature were 110 °C, and the run-time was 12.5 min. Samples were calibrated to certified (±2%) standards of 1% CO_2_ and 2% H_2_ (Calgaz Ltd).

Methane concentrations were analysed using a Carlo Erba HRGC 5160 with a flame ionization detector (FID). A 150 µl aliquot of gas from the exetainers was sampled using a 250 µl SRI gastight valve syringe, and, using the same technique as for hydrogen and carbon dioxide analyses, 100 µl was then injected onto a 30 m × 0.32 mm capillary HP-PLOT/Q with a constant pressure (10 kPa of H_2_). The column temperature was 35 °C, the injector temperature was 150 °C, and the detector temperature was 250 °C. The run-time was 3 min. Calibration was performed by serial dilution of a certified 1500 ppm (±5%) CH_4_ standard (BOC).

Hydrogen and carbon dioxide concentrations were converted to micromoles per microcosm (containing on average 8.3 mL headspace and 4.0 mL liquid at the end of the experiment). Carbon dioxide concentrations were converted to total inorganic carbon (TIC) concentrations using Henry’s Law and carbonate equilibria equations (Supplemental Equations). An ANOVA and a post hoc Tukey test were performed to compare the TIC concentration in the different conditions with each other. An ANOVA was performed to compare the hydrogen concentration in the different conditions with each other.

### Scanning electron microscopy

In the second experiment, samples of the kerogen-rich rock powder after microbial growth were prepared for scanning electron microscopy (SEM). Samples were fixed in a solution of 3% glutaraldehyde in 0.1 M sodium cacodylate buffer (pH = 7.3) for 2 h. The samples were then centrifuged for 10 min at 4,000 g, after which the supernatant was discarded. They were then washed 3 times for 10 min in 0.1 M sodium cacodylate buffer on a rotator, and samples were vortexed for 5 s prior to each of the 10-min rotations. After discarding the buffer for the third time, the samples were postfixed in 1% osmium tetroxide in 0.1 M sodium cacodylate buffer, by vortexing the samples for 5 s and storing them on a rotator for 45 min. Hereafter, the samples were centrifuged for 10 min at 4,000 g and the supernatant was discarded. A further 3 times 10-min washes were performed in 0.1 M sodium cacodylate buffer on a rotator. After each 10-min wash, the samples were centrifuged for 10 min at 4,000 g and the supernatant was discarded, after which new buffer was added, and the samples were vortexed for 5 s. Hereafter, the samples were dehydrated in graded concentrations of ethanol (50%, 70%, 90%, and 3 times 100%) for 10 min each on a rotator. After each 10-min wash, the samples were centrifuged for 10 min at 4,000 g and the supernatant was discarded, after which new ethanol was added, and the samples were vortexed for 5 s. This was followed by critical point drying using liquid carbon dioxide. After mounting the samples on aluminium stubs with carbon tabs attached, the specimens were sputter coated with 20 nm gold palladium and viewed using a Hitachi S-4700 scanning electron microscope.

## Supplementary Information

Below is the link to the electronic supplementary material.

Supplementary Material 1

## Data Availability

The genomic data for this study have been deposited in the Sequence Read Archive (SRA) at the National Center for Biotechnology Information (NCBI) under accession number PRJNA1425804 (https://www.ncbi.nlm.nih.gov/sra/PRJNA1425804).
