# Supplementary material for: Kerogen-rich rocks influence growth and composition of an anaerobic microbial community
Source: Sci Rep. 2026 Mar 8;16:12596. doi: 10.1038/s41598-026-42062-5 (PMC13087281; doi:10.1038/s41598-026-42062-5)
Supplement: Supplementary file 1 — Supplementary Material 1 [file 41598_2026_42062_MOESM1_ESM.docx]

# Supplementary material

**Supplementary Table 1:** bacterial family-level taxa that are only present in kerogen type I.

| **Bacterial family** | **Average abundance in K1 (%)** |
| --- | --- |
| Thiohalorhabdaceae | 1.70 |
| Saprospiraceae | 0.35 |
| Rhodothermaceae | 0.22 |
| Cyclobacteriaceae | 0.13 |
| Hahellaceae | 0.08 |
| Alteromonadaceae | 0.07 |
| Streptomycetaceae | 0.07 |

**Supplementary Table 2:** Description of supplementary raw read fastq files.

| **Sample name** | **Description** |
| --- | --- |
| GC2-B_S107_L001_R1_001.fastq | Starting culture, forward |
| GC2-B_S107_L001_R2_001.fastq | Starting culture, reverse |
| K1A_S89_L001_R1_001.fastq | Kerogen type 1, replicate A, forward |
| K1A_S89_L001_R2_001.fastq | Kerogen type 1, replicate A, reverse |
| K1B_S90_L001_R1_001.fastq | Kerogen type 1, replicate B, forward |
| K1B_S90_L001_R2_001.fastq | Kerogen type 1, replicate B, reverse |
| K1C_S91_L001_R1_001.fastq | Kerogen type 1, replicate C, forward |
| K1C_S91_L001_R2_001.fastq | Kerogen type 1, replicate C, reverse |
| K2A_S92_L001_R1_001.fastq | Kerogen type 2, replicate A, forward |
| K2A_S92_L001_R2_001.fastq | Kerogen type 2, replicate A, reverse |
| K2B_S93_L001_R1_001.fastq | Kerogen type 2, replicate B, forward |
| K2B_S93_L001_R2_001.fastq | Kerogen type 2, replicate B, reverse |
| K2C_S94_L001_R1_001.fastq | Kerogen type 2, replicate C, forward |
| K2C_S94_L001_R2_001.fastq | Kerogen type 2, replicate C, reverse |
| K3A_S95_L001_R1_001.fastq | Kerogen type 3, replicate A, forward |
| K3A_S95_L001_R2_001.fastq | Kerogen type 3, replicate A, reverse |
| K3B_S96_L001_R1_001.fastq | Kerogen type 3, replicate B, forward |
| K3B_S96_L001_R2_001.fastq | Kerogen type 3, replicate B, reverse |
| K3C_S97_L001_R1_001.fastq | Kerogen type 3, replicate C, forward |
| K3C_S97_L001_R2_001.fastq | Kerogen type 3, replicate C, reverse |
| K4A_S98_L001_R1_001.fastq | Kerogen type 4, replicate A, forward |
| K4A_S98_L001_R2_001.fastq | Kerogen type 4, replicate A, reverse |
| K4B_S99_L001_R1_001.fastq | Kerogen type 4, replicate B, forward |
| K4B_S99_L001_R2_001.fastq | Kerogen type 4, replicate B, reverse |
| K4C_S100_L001_R1_001.fastq | Kerogen type 4, replicate C, forward |
| K4C_S100_L001_R2_001.fastq | Kerogen type 4, replicate C, reverse |
| N1_S101_L001_R1_001.fastq | Control 1, replicate A, forward |
| N1_S101_L001_R2_001.fastq | Control 1, replicate A, reverse |
| N2_S102_L001_R1_001.fastq | Control 1, replicate B, forward |
| N2_S102_L001_R2_001.fastq | Control 1, replicate B, reverse |
| N3_S103_L001_R1_001.fastq | Control 1, replicate C, forward |
| N3_S103_L001_R2_001.fastq | Control 1, replicate C, reverse |
| Negative-control_S108_L001_R1_001.fastq | negative control, containing no microcosm sample, forward |
| Negative-control_S108_L001_R2_001.fastq | negative control, containing no microcosm sample, reverse |
| PCR-Neg-16S_S109_L001_R1_001.fastq | negative PCR control sample with no DNA template, forward |
| PCR-Neg-16S_S109_L001_R2_001.fastq | negative PCR control sample with no DNA template, reverse |

**Supplemental Equations:** Carbonate equilibria equations to calculate the total inorganic carbon (TIC) in both the headspace and solution from the measured carbon dioxide concentration in the headspace.

Equation 1: Carbonic acid concentration

$${[H}_{2}{CO}_{3}]= K_{H}\times{pCO}_{2}$$

Equation 2: Bicarbonate concentration

$$[H{CO}_{3}^{-}]= \frac{K_{H}\times K_{1}\times{pCO}_{2}}{{[H}^{+}]}$$

Equation 3: Carbonate concentration

$$[{CO}_{3}^{2-}]= \frac{K_{H}\times K_{1}\times K_{2}\times{pCO}_{2}}{{[H}^{+}]^{2}}$$

Equation 4: Carbon dioxide concentration in the headspace

$${[CO}_{2}]= \frac{V_{H} \times{pCO}_{2}}{V_{m}}$$

Values and abbreviations:

V_H_ = Volume headspace

V_m_ = molar volume of a gas under standard temperature and pressure = 22.4 L

pCO_2_ = partial pressure of CO_2_

Henry’s Law constant of CO_2_ at 25 ºC: $K_{H} = {10}^{-1.5} {mol\times L^{-1}\times atm}^{-1}$

Henry’s Law constant of HCO_3_^-^ at 25 ºC: $K_{1} = {10}^{-6.35} {mol\times L^{-1}\times atm}^{-1}$

Henry’s Law constant of CO_3_^2-^ at 25 ºC: $K_{2} = {10}^{-10.33} {mol\times L^{-1}\times atm}^{-1}$
